# Role of Bisphenol A in the Development and Progression of Colorectal Cancer: Possible Sex-Specific Effects of Endogenous and Exogenous Estrogens

**DOI:** 10.3390/biomedicines13112717

**Published:** 2025-11-05

**Authors:** Elisabetta Iessi, Camilla Cittadini, Francesca Maranghi, Roberta Tassinari, Egidio Iorio, Rossella Puglisi, Gianfranco Mattia, Gianluca Frustagli, Lucia Coppola, Gabriele Lori, Cinzia La Rocca, Daniele Marcoccia, Marta Mollari, Flavia Silvia Galli, Maria Teresa Martino, Cosima Chiapperino, Laura Trilli, Pierpaolo Toto, Alessia Sgroi, Sara Di Matteo, Davide Brocco, Nicola Tinari, Elena Ortona, Paola Matarrese

**Affiliations:** 1 Center for Gender-Specific Medicine, Italian National Institute of Health, 00161 Rome, Italy; camilla.cittadini@iss.it (C.C.); francesca.maranghi@iss.it (F.M.); roberta.tassinari@iss.it (R.T.); rossella.puglisi@iss.it (R.P.); gianfranco.mattia@iss.it (G.M.); lucia.coppola@iss.it (L.C.); gabriele.lori@iss.it (G.L.); cinzia.larocca@iss.it (C.L.R.); elena.ortona@iss.it (E.O.); 2 Core Facilities Service, Italian National Institute of Health, 00161 Rome, Italy; egidio.iorio@iss.it (E.I.); gianluca.frustagli@iss.it (G.F.); 3 Istituto Zooprofilattico Sperimentale del Lazio e della Toscana, Via Appia Nuova 1411, 00178 Rome, Italy; daniele.marcoccia@izslt.it (D.M.); marta.mollari-esterno@izslt.it (M.M.); flavia.galli@izslt.it (F.S.G.); 4 Center for Advanced Studies and Technology (CAST), Università degli Studi “G. d’Annunzio” Chieti–Pescara, via Luigi Polacchi 11, 66100 Chieti, Italy; mtmartino@virgilio.it (M.T.M.); cosima.chiapperino@asl2abruzzo.it (C.C.); lauratrilli99@gmail.com (L.T.); pierpaolototo@gmail.com (P.T.); alessia.sgroi@libero.it (A.S.); sara.dimatteo@outlook.it (S.D.M.); davide.brocco@unich.it (D.B.); ntinari@unich.it (N.T.)

**Keywords:** colorectal cancer, Bisphenol A, 17β-estradiol, silibilin, sex differences

## Abstract

**Background**: Colorectal cancer (CRC) is more prevalent in men, and premenopausal women have a better prognosis than both men and postmenopausal women, suggesting a protective effect of estrogen. Humans are exposed to estrogen-like contaminants such as bisphenol A (BPA), a chemical used in the production of plastics that has been linked to hormone-related malignancies (e.g., breast, ovarian, and prostate cancers). The natural flavonolignan compound silibinin (SIL), acting as an estrogen agonist, may play a protective role in CRC in one or both sexes. **Objectives**: To explore the possible association between BPA and CRC, focusing on its potential pro-tumor role and possible gender differences. Analyzing the possible protective effects of SIL on the development of CRC is the secondary objective of the project. **Methods**: To shed light on the interaction between sex and estrogens, both endogenous and exogenous, in the onset of CRC. To this end, we combined ex vivo, in vitro, and in vivo approaches to deepen our understanding of the molecular mechanisms involved. **Conclusions**: The data provided by this study will contribute to understanding the role of estrogens and their receptors in the onset and progression of CRC and the potential protective role of SIL in both sexes.

## 1. Introduction

According to the date from the World Health Organization, colorectal cancer (CRC) is one of the most common types of malignancy and the second leading cause of cancer death in the world (https://www.who.int/news-room/fact-sheets/detail/colorectal-cancer, accessed on 30 June 2025) [1,2,3]. Epidemiological studies reported that men display a higher prevalence of CRC. Women aged 18–44 with CRC had a better prognosis than men of the same age and postmenopausal women, suggesting a contribution of sex and sex hormones in determining differences in CRC incidence and outcomes [4,5,6]. Additional risk factors, i.e., aging, hereditary predisposition, obesity, sedentary behavior, high consumption of fat and red and processed meat, alcohol, and tobacco were associated with different CRC incidence, diagnosis, treatment, and response to therapies between men and women. Therefore, the interest in the potential role of sex and sex hormones in colon oncogenesis increased over the years.

Sex hormones (progesterone, testosterone, and the most potent estrogen, 17-beta estradiol (E2)) are mainly produced in the gonads but their production could also occur in other peripheral tissues, such as colon. Colon cells can metabolize sex hormones, particularly estrogens, which could influence the development of CRC [7,8]. Previous studies correlated sex steroids and colon physiology and pathophysiology. Among sex hormones, estrogens (in particular E2) seem to exert a protective effect against CRC progression. The biological effects of estrogens are mediated by specific nuclear receptors (estrogen receptors, ERs). ERs belong to the steroid hormone superfamily of nuclear receptors, i.e., mainly detectable in the cell nucleus. However, ERs have also been found in the cytoplasm, at the mitochondrial and plasma membrane level. Two different types of ERs have been identified: the estrogen receptor alpha (ERα), and the estrogen receptor beta (ERβ). In humans, the ERβ is 530 amino acids in length, with a molecular weight of 59 kDa (ERβ1). To date, six different isoforms of ERβ (ERβ1-6) are known. Yet, the full-length size of ERα is 595 amino acids with a molecular weight of 66 kDa (ER66). In the last few years, three further shorter isoforms (at 46, 36 and 30 kDa) have been characterized. Generally, ERα has higher expression in the early stages of CRC and exerts a tumor-promoting role. Indeed, its activation following E2 stimulation up-regulates genes associated with cell proliferation involved in the Wnt/β-catenin signaling pathway. By contrast, ERβ functions as a tumor protector in CRC development, inducing apoptosis and inhibiting tumor growth. Its expression tends to decrease during CRC progression versus malignancy [9,10]. Interestingly, when they are co-expressed, they can heterodimerize and, thus, ERβ reduces the ERα transcriptional activity. ERβ2 and ERβ5 isoforms, which lack the transactivation activity, were identified in primary colorectal carcinoma samples where they heterodimerize with ERβ1, inhibiting its tumor suppressor signaling pathway [11]. Like the full-length ERα, the other shorter isoforms ERα-36 and ERα-46 seemed to be involved in CRC progression through activation of survival signaling pathways like PKC, ERK-MAPK, and PI3K-Akt [12,13]. Therefore, ERα and ERβ play an important role in the pathogenesis of CRC. Indeed, an association was observed between overexpression of the ERβ1 and increased survival in CRC while a down-regulation of ERβ1 expression is associated with a poorer prognosis. In general, estrogen furthers colonic proliferation, binding to ERα, and promotes anti-proliferative and pro-apoptotic effects once linked to ERβ [14,15,16]. Such evidence encourages the use of ERβ-selective agonists for the management of different tumors, including CRC. Once E2 binds to ERs on the cell surface, ERs homodimerize and translocate to the nucleus where they bind to specific DNA response elements (EREs) [17,18], inducing the activation of downstream target genes. The literature data reported that ERα favors the expression of genes associated with cell proliferation, while ERβ up-regulates genes controlling cell cycle progression and apoptosis [19,20]. Interestingly, E2 can differently modulate the ERα and ERβ levels in CRC. In depth, it has been reported that E2 is able to inhibit ERα expression while up-regulating the expression of ERβ through the activation of the p38/MAPK pathway [21,22]. Therefore, the biological effects of E2 in CRC seemed to be dependent on E2 binding to ERs or in the ratio of ERα to ERβ [23]. Changes in the ERs ratio has also been reported [24]. Indeed, a study involving an APC mouse model correlated the E2 inhibition of tumor formation with an increase in ERβ and a decrease in ERα expression in the target tissue [24,25]. Therefore, it would appear that the level of ERs expression, the ratio of expression of the two receptors, and the regulation of their activity by E2, influences the clinical outcome of CRC.

Several natural compounds derived from plants, known as phytoestrogens, act as estrogen agonists, selectively binding ERβ, potentially displaying estrogen-like effects [26]. Among them, silibinin (SIL), the main active component of silymarin extracted by the milk thistle (Silybum marianum), was approved in 2008 by the China Food and Drug Administration for its hepatoprotective effects [27,28]. Notably, SIL, available as a dietary supplement, has gained attention for its anti-inflammatory and anti-tumor effects against diverse cancers, including CRC [28,29,30,31], making it a promising candidate as a potential therapeutic agent in CRC treatment. Indeed, recent studies highlighted its direct anti-tumor effects, its ability to enhance the efficacy of other anticancer drugs, and its potential to improve patient outcomes in CRC [29,30,32,33]. We have observed that SIL once linked to ERβ promotes apoptosis and autophagy, and blocks tumor cell proliferation in lymphoma cells [28]. Interestingly, studies in the literature highlighted the ability of SIL to enhance the expression of ERβ in colon samples [34,35].

The exposure to environmental estrogen-like contaminants has been associated with the onset and progression of several tumors [36]. In particular, bisphenol A (BPA) is a chemical used in the production of polycarbonate plastics and epoxy-phenolic resins for several consumer products (e.g., food contact materials, paints, and thermal paper), leaching to environment and food under particular physical–chemical conditions [37,38,39]. BPA has been recognized as an endocrine disruptor able to interact with several receptors, including ERα, ERβ, and the estrogen-related receptor gamma (ERR), as well as the membrane-bound G protein-coupled estrogen receptor (GPER) [40]. Growing evidence showed BPA effect on mechanism of action related to malignant hormone-related tumors, such as breast [41], ovarian [42], prostate [43], and CRC [44,45,46]. These studies addressed the potential pro-tumoral role of BPA, highlighting different molecular signaling pathways, which could affect cancer progression both in vitro and in vivo. In further details, in CRC, BPA, although structurally and functionally similar to E2, acts directly via steroid hormone receptors, interacting with ERβ with a lower affinity than E2 and behaving as an E2 antagonist by blocking ERβ activities [47,48]. Indeed, growing evidence demonstrated that BPA is able to promote cell proliferation, motility, invasion, and metastasis through induction of epithelial-to-mesenchymal transition by activating the ERK pathway, reducing E-cadherin, and enhancing 5-HT3 receptors, Snail, N-cadherin, and vimentin in CRC [45,49]. In CRC, BPA can also induce DNA damage and mutations favoring oxidative stress conditions [45]. Additionally, BPA can down-regulate apoptosis [50]. Several biomonitoring studies demonstrated that humans are exposed to BPA [50]. Background BPA level was also established for an Italian population [51,52].

However, to our knowledge, human studies exploring the possible association between human exposure to BPA and CRC are limited. The incidence of CRC is partially driven by the impact of lifestyle and behavior risk factors [53]. These risk factors affected also the exposure to endocrine disruptors as well as sex differences leading to a different scenario between women and men [54]. Therefore, the evaluation of exposure considering disaggregated data on sex and gender is recommended [55]. With this respect, the assessment of biomarkers in biomonitoring studies has received more attention to enhance the biological plausibility between internal exposure and adverse health outcomes or diseases [56].

The main objective of this project is to correlate—through a clinical study enrolling people with CRC of both sexes—the exposure to exogenous estrogens and/or estrogen-like environmental contaminants, such as BPA, with the onset and/or progression of CRC. We hypothesized that such treatments (a) interfere with therapy efficacy, relapse, and patient survival, and (b) induce a different response in CRC depending on sex. We will then address whether estrogens and/or BPA (a) would interfere with the efficacy of therapy, relapses, and patient survival, and (b) would induce a different response in CRC according to sex.

Through in vivo studies on murine models, we will evaluate the impact of BPA exposure on the onset of CRC, as well as the potential protective/preventive effects of SIL in male and female C57Bl/6J mice fed the high-fat Western diet, known to promote the development of CRC.

Furthermore, through in vitro studies on 2D and 3D cellular models of human colon cancer cells, we will try to identify the mechanisms by which BPA may promote the development of CRC and the role played by estrogens and their receptors.

The strength and innovation of this project lies in the combination of different approaches: ex vivo analyses on biological samples derived from male and female CRC patients, the use of an animal model of CRC, and 2D and 3D in vitro models for the analyses of the biochemical pathways involved.

The knowledge gained from the implementation of this project will contribute to a better understanding of (i) the role played by an endocrine disruptor, such as BPA, on the onset and progression of colorectal cancer (CRC), (ii) the potential ability of SIL to counteract the effects induced by BPA exposure, and (iii) the identification of one or more sex-specific biomarkers associated with disease severity useful for the clinical management of the disease.

## 2. Aims of the Study

Ex vivo: To evaluate the effects of the exposure to BPA on the onset and/or evolution of CRC. Clinical and biological parameters will be analyzed considering sex, health status, and dietary intake that may help at identifying one or more sex-specific biomarkers associated with disease severity useful for the clinical management of the disease.

In vivo: To assess the contribution of BPA to the onset of CRC and the potential protective effects of SIL in C57Bl/6J mice of both sexes exposed to a standard or Western diet. To this end, by considering sex-related susceptibility to treatments, we will evaluate the expression of specific biomarkers in the gut, plasma, and blood that may be transferable to human study. The sex-specific biomarkers we will select for our analyses will be microRNAs whose expression is modulated by estrogen and/or present on the X chromosome.

In vitro: To dissect the molecular mechanisms underlying the pro-cancer effects of BPA and the protective effects of SIL in presence or absence of E2. Human colon cancer cell lines will be exposed or not exposed to BPA, SIL, E2, or their combination, and the pathways of death, survival, growth, invasiveness, metastasis, and epithelium–mesenchymal transition will be analyzed.

## 3. Methodology and Study Design

This study will integrate different approaches: (i) a clinical study enrolling men and women affected by colorectal cancer (at least 60 subjects per sex) with an equal number of age-matched healthy subjects; (ii) in vivo model of CRC using male and female C57Bl/6J mice, and (iii) 2D and 3D in vitro models using human colon cancer cell lines (HT-29, HCT-116, SW480, SW620), also engineered to overexpress ERβ.

### 3.1. Human Study

To evaluate the possible role of BPA as exogenous estrogen, a case control study will be performed by enrolling women and men affected by CRC (cases) matched with healthy subjects (as controls) for age and sex. A total of 240 subjects (60 per sex per group), determined through a G-power calculation, will be recruited by the Oncology Clinic of the SS. Annunziata Hospital in Chieti. All the patients consecutively referred for CRC will be considered for the enrollment according to the following categories: (a) patients with stage II surgically removed tumor; (b) patients with stage III surgically removed tumor candidate to adjuvant chemotherapy; and (c) patients with metastatic tumors that will be treated with systemic therapy (chemotherapy and biologic therapy or immunotherapy, as appropriate). Enrolled people will be asked to sign a written informed consent to take blood and urine samples, and to fill in a specific questionnaire. The questionnaire will be split into two parts, the first of which will focus on entering demographic data, and the second on questions relating to eating habits. More specifically, you will need to report the frequency and quantity of the specific dishes indicated (where possible). A univocal alphanumeric code will be assigned to each enrolled subjects to ensure data anonymization.

Healthy subjects and patients with a histological diagnosis of colon cancer and able to provide informed consent to participate in the study will be considered eligible.

Subjects with prior or concurrent cancer, and those who underwent radiotherapy or chemotherapy within 5 years prior to enrolment, or in the case of concurrent estrogens or systemic therapies, will be excluded from the study.

A panel of biomarkers in CRC development and progression linked to BPA exposure will be evaluated. The BPA levels and metabolomics profiles (alanine, 3-aminoisobutyrate ascorbate, citrate, creatinine, glycerol, hippurate, taurine threonine, urea, and valine) will be analyzed in urine samples.

Sera samples will be collected and analyzed for specific epithelial/mesenchymal parameters (N- and E- cadherins), hormone levels (17β-estradiol, hydroxy estrogen/16alpha-hydroxyestrone), and inflammatory interleukins.

To evaluate the impact of lifestyle and dietary habits related to BPA exposure and colon cancer risk factors and the possible sex/gender related differences, a structured questionnaire specifically modified for women and men will be designed and administered to the participants. The questionnaire will be adapted from a questionnaire previously used within the LIFE PERSUADED project [57]. A paper as well as an online version of the questionnaire will be created. The online version will be developed based on the paper version of the questionnaire using open-source software, specifically the well-known LAMP framework (HTML + Javascript + PHP + MySQL + Apache) through an interactive online form interfaced with a database, to support the data entry and consistency checking process as well as data processing. Indeed, given that it is necessary to collect questionnaire data in a software-processable file and given the large number of fields in the questionnaire (more than 800), manually creating such a file would be excessively error-prone. The electronic (online) version of the questionnaire allows for guided data entry, which is then automatically serialized into a file. For each enrolled subject (CRC patient or control), identified by a unique alphanumeric code, the data derived from the administered questionnaire, entered online at the time of enrollment, and data derived from the analyses of the biological samples, will be downloaded in an Excel file and will be subjected to statistical analyses. In addition, a digital database will be kept up to date over time where, owing to data obtained from patient medical records, it will be possible to track key values relating to enrolled patients, such as satellite instability, tumor staging, menopausal status, or the use of hormone therapy. Biological samples—blood and urine—will be collected from enrolled patients and healthy volunteers (controls). We will need two tubes with EDTA (purple cap) and one tube without anticoagulant (yellow cap). The latter will be delivered to the CAST molecular oncology laboratory of Chieti (CH) where they will be processed and stored. Additionally, patient tissue slides will be requested from the hospitals where they underwent surgery. Ideally, 10 slides for a patient will be needed, polarized with a positive charge, one of which must be stained with hematoxylin and the others in blank. The slides must be at least 4 microns thick.

Biological materials such as urine, blood, and tissues from patients enrolled at the Oncology Clinic of the SS. Annunziata Hospital in Chieti will be processed and stored for chemical–biological and molecular analyses. In particular, miRomic profiles, qualification of BPA, analyses of ERs, and tumor progression markers will be performed. Blood collected from patients will be placed in two tubes containing EDTA and one tube without EDTA. The tube will be gently inverted several times to ensure homogeneous mixing of the blood and EDTA. The samples will be centrifuged at 2000 rpm for 5 min to separate the blood components based on their density. Serum and plasma, being lighter, will settle at the top of the tube. After centrifugation, the plasma and serum will be collected and aliquoted into new eppendorfs. The aliquots will be stored in freezer boxes at −80 °C for subsequent analyses. The urine collected from the patient in the appropriate sterile container will be aliquoted into two tubes. The aliquots will be stored in the refrigerator at −20 °C for subsequent analysis. Simultaneously with the sample collection and storage process, a detailed database containing all patient and sample information was created to facilitate data access and analysis. This database includes patient personal data (name, surname, and date of birth) and specific details of the collected samples (collection date, sample type, and identification code).

Study on patients will be followed according to the rules of Good Clinical Practice and recommendations of the international guidelines on colon cancer. Ethical aspects will be evaluated by the Ethics Committee of ISS and SS Annunziata Hospital. All enrolled people will be provided with complete information about the study and asked to sign a written informed consent.

### 3.2. Animal Studies

The animal study will be performed in compliance with the EU Council Directive on the welfare of experimental animal 2010/63/EU, the Italian Legislative Decree n. 26 of 4 March 2014 and checked by National Centre for Animal Experimentation and Welfare of ISS. All the relevant information will be recorded for each test group. The number of mice/sex/group will be calculated according to the indications of the OECD Guidelines for testing chemicals (https://www.oecd.org/en/publications/2018/06/test-no-408-repeated-dose-90-day-oral-toxicity-study-in-rodents_g1gh2931.html, accessed on 30 June 2025); the G*Power 3.1.9.7 program will be used to design the study and animal numbers will be calculated using the levels of the proinflammatory cytokine Interleukin-6 (IL-6) as a key endpoint [58].

Adult healthy C57Bl/6J mice of both sexes (body weight 18–25 g, age 7–8 weeks) will be purchased from Envigo (Udine, Italy) and fed with Standard Diet or Western Diet, known to trigger and sustain the early phases of tumorigenesis in mouse colon. Upon arrival, mice will be housed in the same room, in transparent Plexiglas cages and kept under standard laboratory conditions at the animal facility of ISS. After one-week acclimation period, same-sex mice will be randomly allocated to experimental groups, as follow:

Group 1 = fed with Standard Diet;

Group 2 = fed with Western Diet;

Group 3 = fed with Western Diet plus BPA at 2 mg/kg bw per day [59];

Group 4 = fed with Western Diet plus Silibilin 50 mg/kg bw per day;

Group 5 = fed with Western Diet plus Silibilin + BPA.

Mice will be treated for 3 (T1, Intermediate sacrifice) and 6 months (T2) in the diet.

The dose levels of BPA, 2 mg/kg bw per day, was selected based on estimated exposure levels for the Italian pediatric population. Moreover, an in vivo study on juvenile rodent models observed a significant up-regulation of IL-6 gene expression, a key biomarker associated with CRC [59]. SIL dose level, 50 mg/kg body weight per day, was chosen based on the literature data reporting its protective effects against CRC in animal models [60]. Animals will be checked daily for health status, body weight, and feed consumption recorded once a week. At T1 (to evaluate early markers of CRC induction) and T2, mice will be anesthetized with gaseous solution of isofluorane, blood will be collected by cardiac puncture, and mice will be humanely killed by exhalation of CO2.

The colorectal tissue will be removed from all mice, its length measured, and fixed as follows:

- One portion in liquid nitrogen and stored at −80 °C for ER gene expression analysis;
- One portion in 10% formalin for histopathological analysis.

Parameters will be assessed as follows:

- General toxicology: animal weight, feed consumption, organ weight, and colorectal length;
- Clinical serum biochemistry: biomarkers of liver, kidney, and metabolic toxicity using an automated veterinary spectrophotometer;
- Specific serum biomarkers of CRC onset: N-CAD, E-CAD, and IL6 using ELISA tests with mouse-specific kits;
- Histopathological analysis of the colorectal tissue;
- ERα and ERβ gene expression in the colorectal region by real-time PCR.

Total RNA from the colorectal tissue of at least three mice per group will be extracted for small-RNA profiling by NGS. MiRomic analysis will indicate some miRNA–disease associations in the different groups of mice, leading to identifying possible early, sex-specific biomarkers of precancerous CRC onset.

### 3.3. In Vitro Studies

We will use 2D and 3D cell cultures models. The 3D in vitro model will be used as a more representative model of the in vivo physiology of the CRC. According to the results obtained from the 2D cell lines, we will select the cell line to use for the 3D model to recapitulate the in vivo model. We will use the human colon cancer cell lines HT-29, HCT-116, SW480, and SW620, which do not express estrogen receptor beta. Two-dimensional cell lines will be cultured in RPMI (Gibco, Milan, Italy), supplemented with 10% fetal bovine serum (FBS), 100 U/mL penicillin, 100 μg/mL streptomycin, and 2 mM L-Glutamine. Cells will be maintained in an incubator at 37 °C, 5% CO2, and 90% humidity, and they will be used between passages 3 and 10. All experiments will be performed in RPMI without phenol red (Gibco, Milan, Italy), supplemented with 5% serum charcoal-stripped serum (CS-FBS), 100 U/mL penicillin, 100 μg/mL streptomycin, and 2 mM L-Glutamine. The concentrations of BPA and SIL to be used in our experimental settings will be chosen based on the literature data. Dose–response curves will also be performed. To strengthen the interpretation of the results, we will include a reference control represented by a non-transformed human colonic cell line, derived from ATCC, the immortalized CCD-841 CoN adherent cell line, isolated from colon tissue of a healthy donor (ATCC reference number CRL-1790). Cells will be then treated with BPA and/or SIL, alone or in combination with E2 or the ERβ-selective agonist ERB 041 or the ERα36-selective agonist IC162 for 48 h or 72 h. Supernatants and cell pellets will be harvested for gene and protein expression analysis of estrogen receptors and matrix metalloproteinases (MMP-2 and -9) as well as vimentin and E-cadherin and a panel of inflammatory interleukins using q-PCR and/or Western blot (WB) techniques and ELISA assays, as described below. In addition, the cultures will be subjected to functional assays, such as cell proliferation, viability, apoptosis, and autophagy using either CellTiter 96 AQueous One Solution Cell Proliferation Assay, CyQUANT Cell Proliferation Assay and Caspase-1/3/7 Assay, CYTO-ID^®^ Autophagy detection kit and/or WB, respectively. We will analyze autophagic markers, such as p62 and LC3, based on our previous work, where we demonstrated that autophagy is one of the key mechanisms in ERβ-mediated tumor growth inhibition [28,61,62]. Moreover, a recent study conducted on pancreatic cells showed that BPA could induce autophagy by regulating the PTEN/PI3K/AKT/mTOR pathway [63].

For migration assays, transwell inserts will be used with 8-microm pore size. Cells that reach the underside of the filter will be stained with Diff-Quik staining set. For cell invasion assays, transwell inserts with 8-micron pore size will be coated with Matrigel.

## 4. Planned Analyses

### 4.1. Metabolomics Profiles by NMR Spectroscopy and BPA Quantification

The BPA levels and metabolomics profiles will be analyzed in urine samples by nuclear magnetic resonance NMR spectroscopy (14.1T NMR instrument), at Core Facilities of ISS. For each sample, two monodimensional 1H NMR spectra will be acquired as follows: (1) a standard NOESY 1Dpresat to obtain a spectrum in which signals of both metabolites and high molecular weight molecules are visible, and (2) a standard CPMG pulse sequence, designed for the selective observation of small molecule components in solutions containing macromolecules. Quantification of the analyses will be performed by dedicated software and multivariate analysis. For metabolite assignment, an internal NMR spectral library of pure organic compounds, public databases such as the Human Metabolome Database, stored reference NMR spectra of metabolites, and the literature data will be used.

### 4.2. Biomarker Analyses

Urine and/or sera of mice and human, as well as cell culture supernatants (SN), will be collected and analyzed for specific epithelial/mesenchymal parameters (i.e., N and E cadherins), 17ß-estradiol, hydroxyestro-gen/16alpha-hydroxyestrone, and inflammatory interleukins using commercial ELISA assays.

### 4.3. Immunohistochemical (IHC) Analysis for Detection of Estrogen Receptors

The expression of ERα, ERα36, and ERβ in surgically removed cancer tissue will be assessed on primary cancer specimens by IHC in deparaffinized tissue slides. ERα, ERα36, and ERβ expression will also be evaluated in tumor samples of mice. To this aim, the large intestine of both treated and control mice or patients will be excised, divided, and stored as follows: flash frozen in liquid nitrogen and stored at −80 °C, fixed in formalin, embedded in paraffin, cut into 5-micron sections, and stained with hematoxylin and eosin for light microscopy analysis with different lenses. The histopathological and morphometric analysis (Villus height/Crypt depth) will be then performed. After incubation with the primary antibodies, the sections will be processed using Dako Real EnVison kit. A three-level scoring system will be applied that involves the staining intensity as well as the percentage of positivity in the cancer cells. Tumors will be regarded as negative for ERβ/ERα/ERα36 expression, if <10% of the cells show positive staining. A moderate expression will be defined as weak positive staining of >50% or strong positive staining in 10/50% of the cells. High expression of ERβ/ERα/ERα36 will be assigned if >50% of the cells show strong positive staining.

### 4.4. Analysis of Human and Mouse miRNA Expression Profile

The global miRNA expression profile will be examined by analyzing plasma samples and/or colorectal tissue from both humans and mice. To this end, taking advantage of the general conserved sequence of the main miRNA family, all patients’ miRNA profiles will be matched with miRNA profiles obtained from the mouse model of BPA dependent CRC. Validation will be achieved by miRNA specific molecular and cellular methods. A parallel proteomic analysis will be performed to validate miRNA targeting association.

Small RNA data will be performed by Next-Generation Sequencing after extraction of EDTA containing plasma samples and/or colorectal tissue of both patients and mice. All RNA analysis will be conducted by the Bioinformatic/Microarray Facility Service, Tecnopolo LTTA of Ferrara. Polymerase chain reaction (PCR) will be run to validate the identified miRNAs in the different group of patients or mice. Proteomic data from plasma or tissue of both human tumors and murine colorectal samples will be analyzed by FAST core facilities of the ISS. Results will be matched with DIANA-miRPath 3.0 webserver and TargetScan predictive tool for target prediction of interactions between miRNA and predicted target mRNAs. The enriched pathway analysis will be performed using DAVID (https://davidbioinformatics.nih.gov/, accessed on 30 June 2025) and PANTHER (https://www.pantherdb.org/, accessed on 30 June 2025) tools. The Kyoto Encyclopedia of Genes and Genomes (KEGG: https://www.genome.jp/kegg/pathway.html, accessed on 30 June 2025) database will be selected as a reference set for the pathway analysis. Due to the highly conserved miRNA sequences and functions between mice and humans, the most interesting differentially expressed miRNAs previously quantified in mice and validated by real-time PCR in patient plasma, at various tumor stages, will be evaluated for correct expression in the colon epithelial cells of both mouse and human biopsies by miRNA-specific in situ hybridization.

### 4.5. Protein Determination by Western Blot Analysis

After treatment, 2D and 3D colon cancer cell pellets will be lysed in a lysis buffer containing 1% of NP40, 50 mM Tri-sHCl pH 7.4, 150 mM NaCl, 2 mM EDTA, 50 mM NaF, 0.1% SDS, and 0.5% Na-deossicolato and proteinase inhibitor cocktail. Cell lysates will be subjected to sodium-dodecyl sulfate polyacrilamide gel electrophoresis and transferred to nitrocellulose membranes. Membranes will be blocked with 5% dried milk in TBS containing 0.05% Tween-20. Then, immunoblotting analyses will be carried out using specific antibodies directed against estrogen receptors, vimentin and E-cadherin, known to be involved in epithelial–mesenchymal transition (EMT), p62 and LC3, typical autophagic markers, and matrix metalloproteinases (i.e., MMP-2 and -9). Blots will be treated with appropriate secondary antibodies conjugated with horseradish peroxidase followed by ECL detection. Equal loading of proteins will be verified by immunoblotting with anti-β-actin or GAPDH or HSC70 antibodies. Densitometric analysis will be performed using a molecular Image Lab 5.0 (Bio-Rad, Hercules, CA, USA).

### 4.6. ERβ Overexpression by Lentiviral Infections

The human CRC cell lines HT-29, HCT-116, SW480, and SW620, which do not naturally express estrogen receptor beta (ERβ), will be transduced with an ERβ construct using a third-generation lentiviral system. Lentiviral transduction will be carried out following a multi-step protocol. Specifically, the packaging plasmid pPAX will be used to supply the essential viral proteins, while the envelope glycoprotein will be provided by VSV-G. Additionally, a transfer plasmid encoding the estrogen receptor beta (ERβ) will be included to enable its expression in the target cell lines. This plasmid also contains genes encoding blasticidin (BSD) resistance and green fluorescent protein (GFP), enabling both antibiotic selection and visual confirmation of successful transduction. To produce the lentiviral particles, the three plasmids, pPAX, VSV-G, and the ERβ-encoding transfer plasmid, will be co-transfected into HEK293T cells. The resulting viral supernatant will be used to infect target colorectal cancer cell lines: HT29, SW480, SW620, and HCT116. Transduction efficiency will be evaluated by monitoring GFP expression via flow cytometry, while ERβ overexpression will be assessed using Real-Time PCR and Western blot analysis.

Infection Validation by flow cytometry. GFP fluorescence will be compared across three groups: cells transduced with the ERβ-expressing plasmid, cells transduced with a control plasmid, and wild-type (uninfected) cells. This comparative analysis will enable the evaluation of transduction efficiency as well as the specificity of gene delivery.

Infection Validation by Real-Time PCR Analyses. RNA will be extracted and purified from the infected cells using the MagPurix kit. Subsequently, 1 µg of purified RNA from each sample will be reverse-transcribed into complementary DNA (cDNA) using the iScript™ cDNA Synthesis Kit (Bio-Rad). Each cDNA sample will be amplified in triplicate using the SsoAdvanced™ SYBR^®^ Green Supermix (Bio-Rad) and analyzed on a QuantStudio™ 7 Flex Real-Time PCR System (Applied Biosystem | Thermo Fisher Scientific, Waltham, MA, USA). All quantification cycle (Cq) values are expected to be below 30. Gene expression levels will be normalized to GAPDH, which will serve as the reference gene. At the end of the run, a melting curve analysis will be performed to confirm the specificity of the amplification.

### 4.7. Statistical Methods

We aim at identifying a difference between patients of different CRC stage within sex (3 + 3 comparisons), and differences between sexes within CRC stages (three comparisons), by a Mann–Whitney U test. Setting the experiment-wise two-tail alpha level = 0.05 (corresponding to a pairwise alpha = 0.00555 with Bonferroni’s correction to account for the nine multiple comparisons described above), and power = 0.80, we will need at least 15 subjects per sex/CRC stage to assess a difference between subgroups equal to 1.5 SD (Cohen’s d = 1.5). On this basis, we thus decided to enroll 20 subjects per sex/CRC stage, i.e., a total of 60 male and 60 female patients and a total of 60 male and 60 female healthy subjects to be used as controls.

Statistical analysis will be performed with STATA 16.1 (StataCorp, College Station, TX, USA). A *p*-value < 0.05 will be considered statistically significant.

For quantitative variables, the measures of central tendency (mean, median, and mode) and dispersion (standard deviation and percentiles) will be calculated. The normality of the distribution will be assessed (Kurtosis and Skewness) to define if the inferential statistics can be conducted with parametric or non-parametric tests. For qualitative variables, the frequency and the confidence intervals according to the binomial distribution will be calculated.

Inferential statistical analyses: for quantitative variables, parametric or non-parametric tests will be applied to analyze the correlation between the outcome variables (internal exposure level) and the other predictive variables.

For quantitative variables, Wilcoxon–Mann–Whitney and Kruskal–Wallis, with Dunns post hoc evaluation where applicable, will be used to evaluate significant differences among groups. Multivariate analyses will be applied for urinary metabolites quantification using Metaboanalyst 6.0 (https://www.metaboanalyst.ca/, accessed on 30 June 2025) software tool on binned spectra and/or metabolite concentrations.

Principal component analysis (PCA) will be used as unsupervised exploratory analysis to obtain a preliminary outlook of the data (presence of clusters or outliers). Orthogonal Partial Least Squares Discriminant Analysis (OPLS-DA) will be used to increase supervised data reduction and obtain the best discrimination between the analyzed groups. In order to reduce false discoveries, false discovery rate correction (FDR) will be then applied using the Benjamini and Hochberg method. Univariate and multivariate analyses will be performed to correlate BPA levels in humans, transformed in dichotomous variables according to the percentiles, and the predictive variables (i.e., questionnaire data). The associations between biomarker values and outcome will be evaluated with logistic regression models, and log odds ratios with corresponding 95% confidence intervals will be reported.

For miRNA data analysis, a cutoff of ±2 will be established between samples from different staging of the disease. PCR miRNA expression data will be shown as mean ± standard error of the mean (SEM) from triplicate experiments. Other data significance will be calculated assuming equal standard deviations and variance with a two-tailed Student’s *t*-test.

The results of in vivo study, presented as mean ± standard deviation, will be analyzed with the Wilcoxon test, followed by appropriate pairwise comparisons (Mann–Whitney test). The histological data, presented as quantal data, will be analyzed using the Mid-*p* exact test.

All in vitro data will be obtained from three independent experiments performed in triplicate. Cytotoxicity data will be expressed as mean ± S.D. and will be analyzed by the non-parametric Dunnett test for multiple comparisons (software SAS JMP Statistical Discovery v14.0, Milan, Italy). Differences will be also determined either with two-way repeated measures analysis of variance (ANOVA) with Bonferroni’s multiple comparison test, or by student’s *t*-test, using GraphPad Prism 5 software (GraphPad, San Diego, CA, USA). Gene expression, expressed as arbitrary units upon normalization with the reference housekeeping gene GAPDH, will be expressed as mean ± S.D. and will be analyzed by Student’s *t*-test (software SAS JMP Statistical Discovery v14.0, Milan, Italy). Throughout the study, *p*-values ≤ 0.05, ≤0.01 and ≤0.001 will be considered statistically significant.

## 5. Expected Results

The results of this study will help to better elucidate the possible role of endogenous and exogenous estrogens in prevention or onset and evolution of CRC. We expect to gain a deeper understanding of the molecular mechanisms underlying the pro-cancer effects of BPA and the potential protective effects of estrogens or estrogen mimetic compounds such as SIL.

The results of this study will help to better elucidate the possible pro-cancer role of BPA and of estrogen on the onset and evolution of CRC as a function of sex. We aim to elucidate the molecular mechanisms underlying the pro-carcinogenic effects of BPA, as well as the potential protective roles of estrogens and estrogen-mimetic compounds such as SIL.

Moreover, the project will provide data on new sex-related biomarkers linked to BPA exposure, allowing the identification of possible determinants of exposure in colon cancer.

Finally, the animal study will enhance our understanding of the impact and the mechanisms associated with BPA exposure and spontaneous CRC induction according to sex and dietary habits.

The insights gained from this study hold potential for the foundation of a gender-specific approach in the management of the CRC patient, thus allowing for numerous benefits, such as reduced disability, improved work productivity, and quality of life. The recent literature data supports a causal role of BPA, even at low levels, in the development of many cancers, including that of the colon, and in determining their response to antineoplastic therapy. An in-depth knowledge of the biochemical and molecular mechanisms involved in these effects would be extremely useful for implementing preventive strategies and for improving the clinical and therapeutic management of colon cancer. Furthermore, confirming the role of BPA as a cancer promoter could favor adequate preventive policies by reducing the exposure of the population to this environmental contaminant.

## Abbreviations

The following abbreviations are used in this manuscript:

CRC	Colorectal cancer
E2	17-beta estradiol
ERs	Estrogen Receptors
SIL	Silibinin
ERR	Estrogen-related receptor gamma
GPER	Membrane-bound G protein-coupled estrogen receptor
BPA	Bisphenol A
